# Supplementary material for: Influenza vaccine effectiveness against influenza-associated hospitalizations and emergency department or urgent care encounters among children and adults — United States, 2024–25 season
Source: medRxiv. 2026 Apr 24:2026.04.22.26350853. Preprint. [Version 1] doi: 10.64898/2026.04.22.26350853 (PMC13131709; doi:10.64898/2026.04.22.26350853)
Supplement: Supplement 1 [file media-1.pdf]

Supplemental Table 1. ICD-10 Codes Used to Define Acute Respiratory Illness

**Acute Respiratory Illness Description**	**ICD-10 Codes**
Influenza Pneumonia	J09.X1, J10.0*, J11.0*
Other Viral Pneumonia	J12.* (excluding J12.81 and J12.82)
Bacterial Pneumonia	J13, J14, J15.*, J16.*, J17, J18.*
Influenza Disease	J09.*, J10.1, J10.2, J10.8*, J11.1, J11.2, J11.8* (excluding J09.X1)
Acute Respiratory Distress Syndrome	J80
COPD Exacerbation	J44.1[a]
Asthma Exacerbation	J45.21, J45.22, J45.31, J45.32, J45.41, J45.42, J45.51, J45.52, J45.901, J45.902
Respiratory Failure	J96.0*, J96.2*, R09.2, J96.9*
Other Acute Lower Respiratory Tract Infections	B97.4, J20.*, J21.*, J22, J40, J44.0[a], J41.*, J42, J43.*, J47.*, J85.*, J86.*
Sinusitis	J01.*, J32.*[a]
Acute Upper Respiratory Tract Infections	J00.*, J02.*, J03.*, J04.*, J05.*, J06.*
Acute Respiratory Illness Signs and Symptoms	R04.2, R05, R05.1, R05.2, R05.4, R05.8, R05.9, R06.00, R06.02, R06.03, R06.1, R06.2, R06.8, R06.81, R06.82, R06.89, R07.1, R09.0*, R09.1, R09.2, R09.3, R09.8*

Note: All ICD-10 codes with * include all child codes under the specific parent code.

[a]Denotes ICD codes used only for adults aged ≥18 years.

Supplemental Table 2. Dates of inclusion in influenza vaccine effectiveness analyses by VISION site and care setting[a]

Site	Hospitalizations		Emergency Department or Urgent Care Encounters	
	Start Date	End Date	Start Date	End Date
Site A	10/13/2024	4/29/2025	10/5/2024	4/30/2025
Site B	11/8/2024	4/28/2025	10/3/2024	4/30/2025
Site C	10/8/2024	4/30/2025	10/1/2024	4/30/2025
Site D	10/3/2024	4/29/2025	10/1/2024	4/30/2025
Site E	10/19/2024	4/29/2025	10/3/2024	4/30/2025
Site F	10/1/2024	4/30/2025	10/2/2024	4/30/2025
Site G	10/2/2024	4/30/2025	10/1/2024	4/30/2025

Abbreviations: VISION = Virtual SARS-CoV-2, Influenza, and Other respiratory viruses Network

[a] Influenza vaccine effectiveness analyses included acute respiratory illness-associated encounters from the date of the first influenza-positive case on or after October 1, 2024 by VISION site and setting to the date of the last influenza-positive case on or before April 30, 2025 by VISION site and setting.

Supplemental Table 3. ICD-10 Codes Used to Define Underlying Medical Conditions and Immunocompromising Conditions

**Underlying Medical Condition**	**ICD-10 Codes**
Respiratory Condition	J45.*, J40.[a], J41.*[a], J42.[a], J43.*[a], J44.*[a], D86.0, E88.01, J47.*, J60[a], J61[a], J62.*[a], J63.*[a], J64[a], J65[a], J66.*, J67.0, J67.1, J67.2, J67.3, J67.4, J67.5, J67.6, J67.7, J67.8, J68.*, J70.*, J81.1, J84.*, J95.*, J96.1*, J99., P26.*, P27.*, B39.*, B40.1, B40.2, B41.0, B44.0, B44.1 , B45.*, B46.0, A15.*, A31.0, G47.3*[b], P27.0[b], P27.1[b], P27.8[b], P27.9[b], E84.*, Q33.*[b],Q34.*[b], Q30.*[b], Q31.*[b], Q32.*[b], Q39.*[b], Q79.0[b], J45.901[b], J45.902[b], J45.909[b]
Cardiovascular Condition	I50.*, I21.*, I22.*, I23.*, I24.*, I25.*, I10., I11.*, I13.*, I15.*, I01.*, I02.0, I09.*, I27.*, I28.*, I31.*, I42.*, I43., I44.*, I46.*, I51.0, I51.1, I51.2, I51.3, I51.5, I51.7, I51.8*, I51.9, I52., I97.0, I97.1*, M31.0, M31.1*, M31.2, M31.4, M31.6, M31.7, M31.8, M31.9, Z95.*, Z98.61, I71.*, I72.*, I73.*, I74.*, I75.*, I79.*, I26.*, I05.*, I06.*, I07.*, I08.*, I34.*, I35.*, I36.*, I37.*, I48.*, I50.9, I42.9, Q20.*[a], Q21.*, Q22.*, Q23.*, Q24.*, Q25.*, Q26.*, Q27.0, Q27.3*, Q27.4, Q27.8, Q27.9, Q28.*, P29.30, Q89.3[a]
Cerebrovascular Condition	I60.*, I61.*, I63.*, I62.*, I68.*, I69.*
Neurologic and/or Musculoskeletal Condition	F01.*[a], F02.*[a], F03.*[a], G30.*[a], H49.81*, M12.0*, M36.0, E75.02, E75.19, E75.4, F71., F72., F73., F84.2, G10., G11.*, G12.*, G13.*, G14., G20.*, G21.*, G23.*, G24.*, G25.*, G26., G31.*, G32.*, G35., G36.*, G37.*, G40.*, G45.*, G46.*, G60.*, G61.*, G62.*, G63., G64., G70.*, G71.*, G73.*, G80.*, G81.*, G82.*, G83.*, G90.3, G91.*, G93.*, G94., G95.*, G99.2, P91.*, Q00.*, Q01.*, Q02., Q03.*, Q04.*, Q05.*, Q06.*, Q07.*, Q76.*, Q77.*, Q78.*, Q79.1, Q79.2, Q79.3, Q79.4, Q79.5*, Q79.6*, Q79.8, Q79.9, Q85.*, Q87.4*, Q91.*, Q92.*, Q93.*, Q96.*, R41.*, R53.2, R54., Q90.*, R56.*[b], P90[b], P91.0[b], P52.*[b], E70.*[b], E71.*[b], E72.*[b], E74.*[b], E75.2*[b], E76.*[b], E77.*[b], E78.*[b], E79.*[b], E80.*[b], P70.*[b], P71.*[b], P72.*[b], P74.*[b], P94.*[b]
Hematologic Condition	D55.*, D56.0, D56.1, D56.2, D56.4, D56.5, D56.8, D56.9, D57.0*, D57.1, D57.2*, D57.4*, D57.8*, D58.*, D59.*, D60.*, D61.*, D64.0, D64.1, D64.2, D64.3, D64.4, D64.8*, D65, D66, D67, D68.*
Endocrine Condition	E10.*, E11.*, E08.*, E09.*, E13.*, E00.*, E01.*, E03.*, E05.*, E06.*, E15. , E16.*, E20.*, E21.*, E22.*, E23.*, E24.*, E25.*, E26.*, E27.*, E28.*, E29.*, E31.*, E32.*, E34.*, E70.*[a], E71.*[a], E72.*[a], E74.*[a], E75.2*[a], E76.*[a], E77.*[a], E78.*[a], E79.*[a], E80.*[a], E83.*, E85.*, E88.02, E88.09, E88.1, E88.2, E88.3, E88.4*, E88.8*, E88.9
Renal Disease	I12.*, I13.* N01.*, N02.*, N03.*, N04.*, N05.*, N06.*, N07.*, N08., N11.*, N14.*, N15.*, N16., N18.*, N25.*, N26.*, N28.*, Q27.1, Q27.2, Q60.*, Z49.*, Z91.15*, Z94.0, Z99.2
Gastrointestinal Condition	B18.*, I81, I85.*, K70.*, K71.*, K72.*, K73.*, K74.*, K75.*, K76.*, K77., K50.*, K51.*, K52.*
Other Underlying Conditions	P07.*[b], R62.*[b], Z93.0[b], Z93.1[b], Z99.0[b], Z99.11[b], Z99.81[b], Z99.89[b]

Immunocompromising Conditions	**Hematologic Malignancy**: C81.*, C82.*, C83.*, C84.*, C85.*, C86.*, C88.*, C90.*, C91.*, C92.*, C93.*, C94.*, C95.*, C96.*, D46.*, D61.0*, D70.0, D61.2, D61.9, D71.*<br>**Solid Malignancy**: C00.*, C01., C02.*, C03.*, C04.*, C05.*, C06.*, C07., C08.*, C09.*, C10.*, C11.*, C12., C13.*, C14.*, C15.*, C16.*, C17.*, C18.*, C19., C20., C21.*, C22.*, C23., C24.*, C25.*, C26.*, C30.*, C31.*, C32.*, C33, C34.*, C37, C38.*, C39.*, C40.*, C41.*, C43.*, C45.*, C46.*, C47.*, C48.*, C49.*, C50.*, C51.*, C52, C53.*, C54.*, C55, C56.*, C57.*, C58, C60.*, C61, C62.*, C63.*, C64.*, C65.*, C66.*, C67.*, C68.*, C69.*, C70.*, C71.*, C72.*, C73, C74.*, C75.*, C76.*, C77.*, C78.*, C79.*, C7A.*, C7B.*, C80.*, Z51.0, Z51.1*, C4A.*<br>**Solid Organ or Bone Marrow Transplant**: T86.0*, T86.1*, T86.2*, T86.3*, T86.4*, T86.5, T86.81*, T86.85*, D47.Z1, Z48.2*, Z94.*, Z98.85<br>**Rheumatologic/inflammatory Disorders:** D86.*, E85.1, E85.2, E85.3, E85.4, E85.8*, E85.9, G35.*, J67.9, L40.54, L40.59, L93.0, L93.2, L94.*, M05.*, M06.*, M07.*, M08.*, M30.*, M31.3*, M31.5, M32.*, M33.*, M34.*, M35.3, M35.89, M35.9, M46.0*, M46.1, M46.8*, M46.9*<br>**Other Intrinsic Immune Condition or Immunodeficiency**: D27.9, D72.89, D80.*, D81.0, D81.1, D81.2, D81.4, D81.5, D81.6, D81.7, D81.8*, D81.9, D82.*, D83.*, D84.*, D89.0, D89.1, D89.3, D89.4*, D89.8*, D89.9, K70.3*, K70.4*, K72.*, K74.3, K74.4, K74.5, K74.6*, N04.*, R18.0<br>**HIV/AIDS:** B20., B97.35, O98.7*, Z21, E88.14

Abbreviations: ICD = International Classification of Diseases

Note: All ICD-10 codes with * include all child codes under the specific parent code.

[a]Denotes ICD codes used only for adults aged ≥18 years.

[b]Denotes ICD codes used only for children aged <18 years.

Supplemental Figure 1. Exclusion flow diagrams for hospitalizations and emergency department or urgent care encounters

A) Exclusion flow diagram for hospitalizations

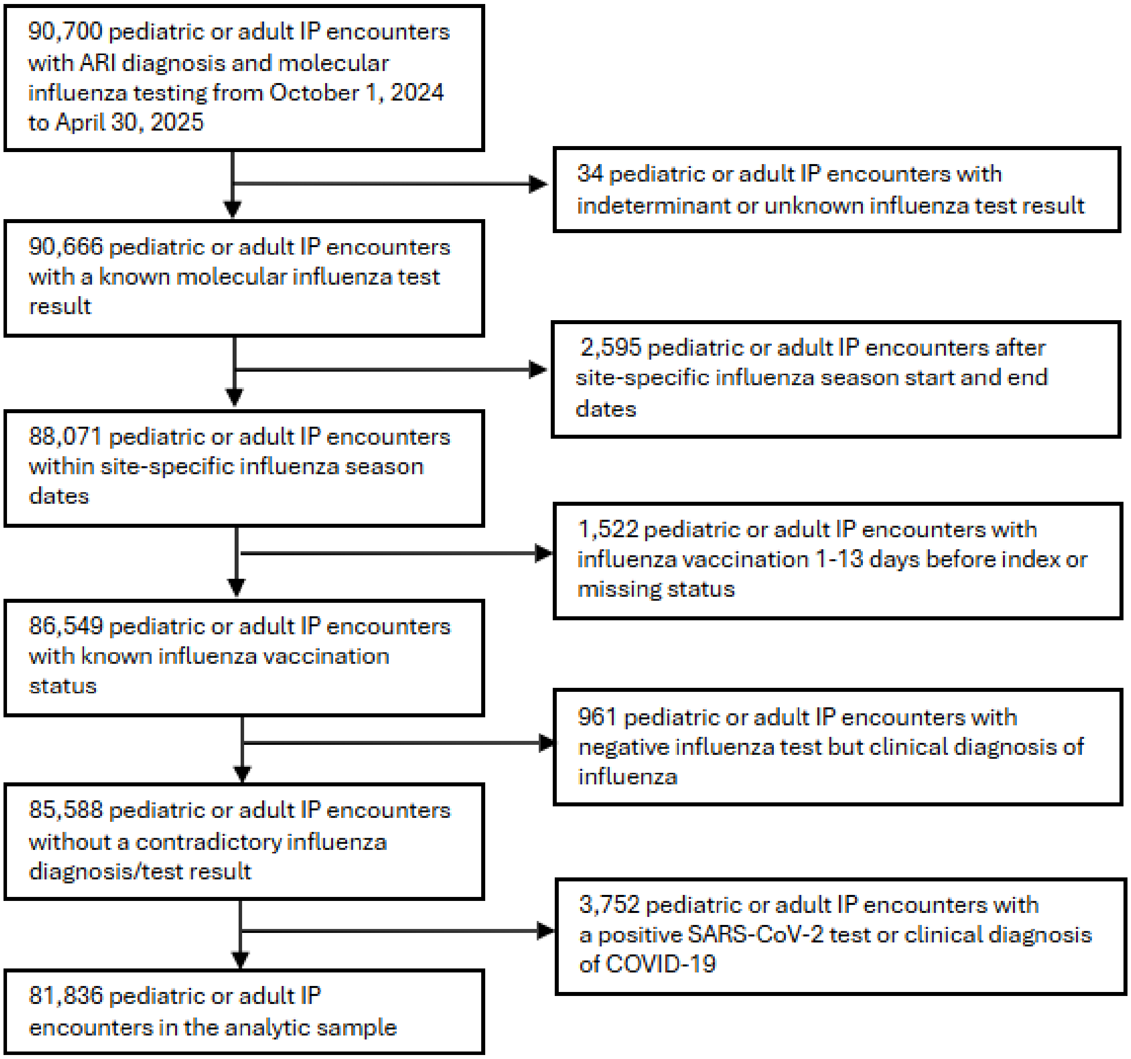

B) Exclusion flow diagram for emergency department or urgent care encounters

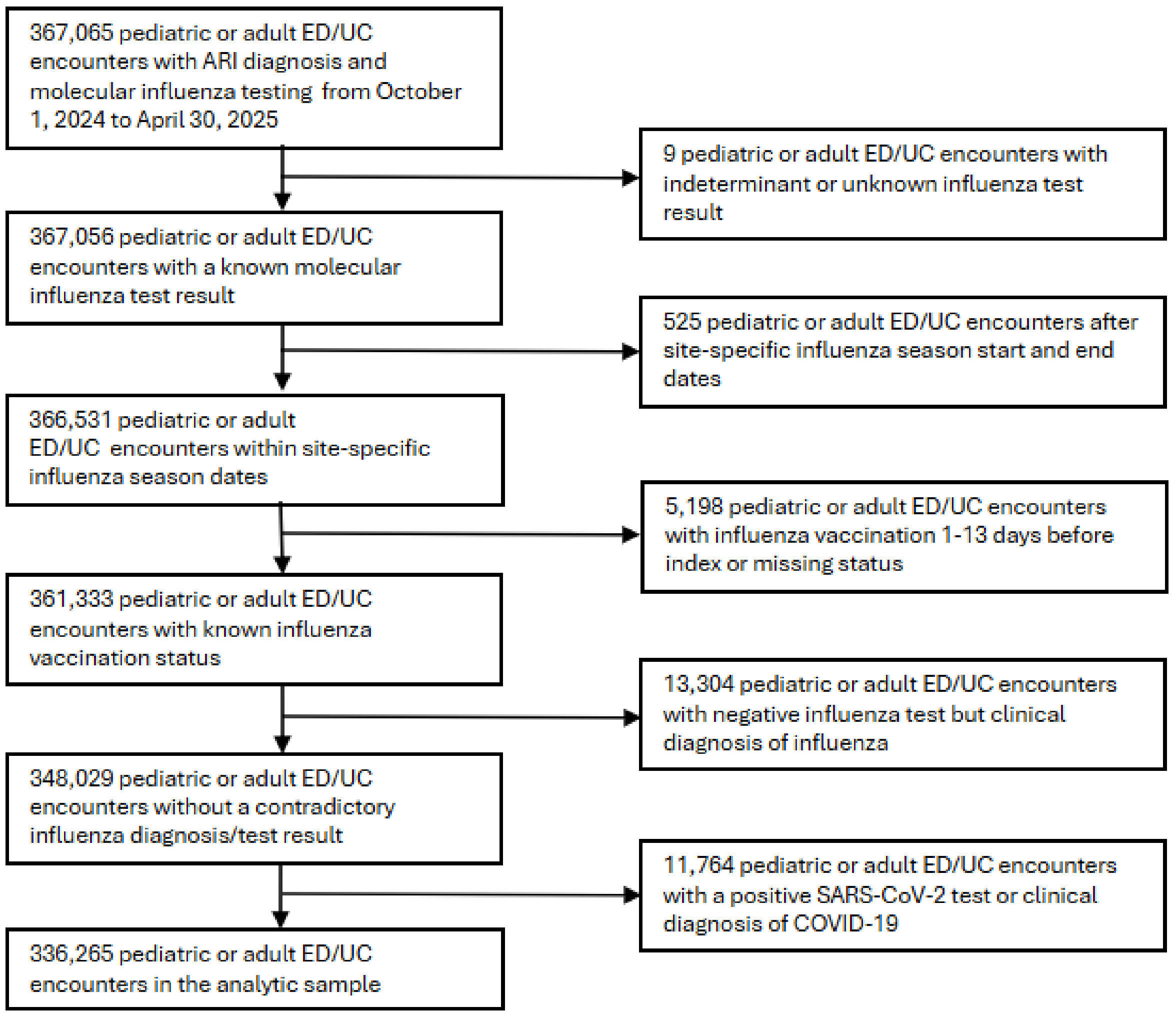

Abbreviations: ARI = acute respiratory illness; ED/UC = emergency department or urgent care; IP = inpatient

Supplemental Figure 2. Influenza-associated medical encounters and percent positivity by week, age group, and setting — VISION, October 2024–April 2025

A) Influenza-positive hospitalizations and percent positivity by week among children aged 6 months–17 years

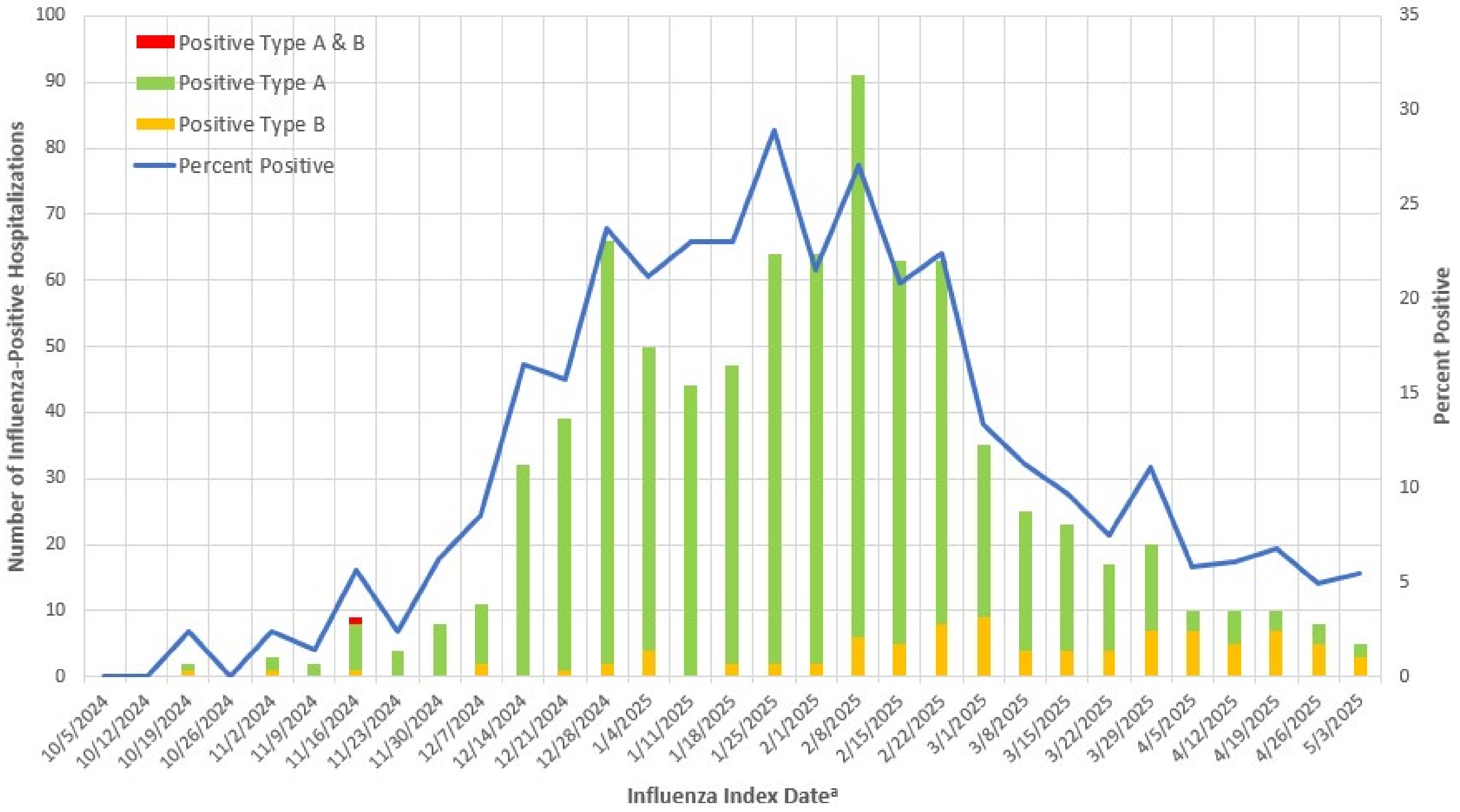

B) Influenza-positive emergency department or urgent care encounters and percent positivity by week among children aged 6 months–17 years

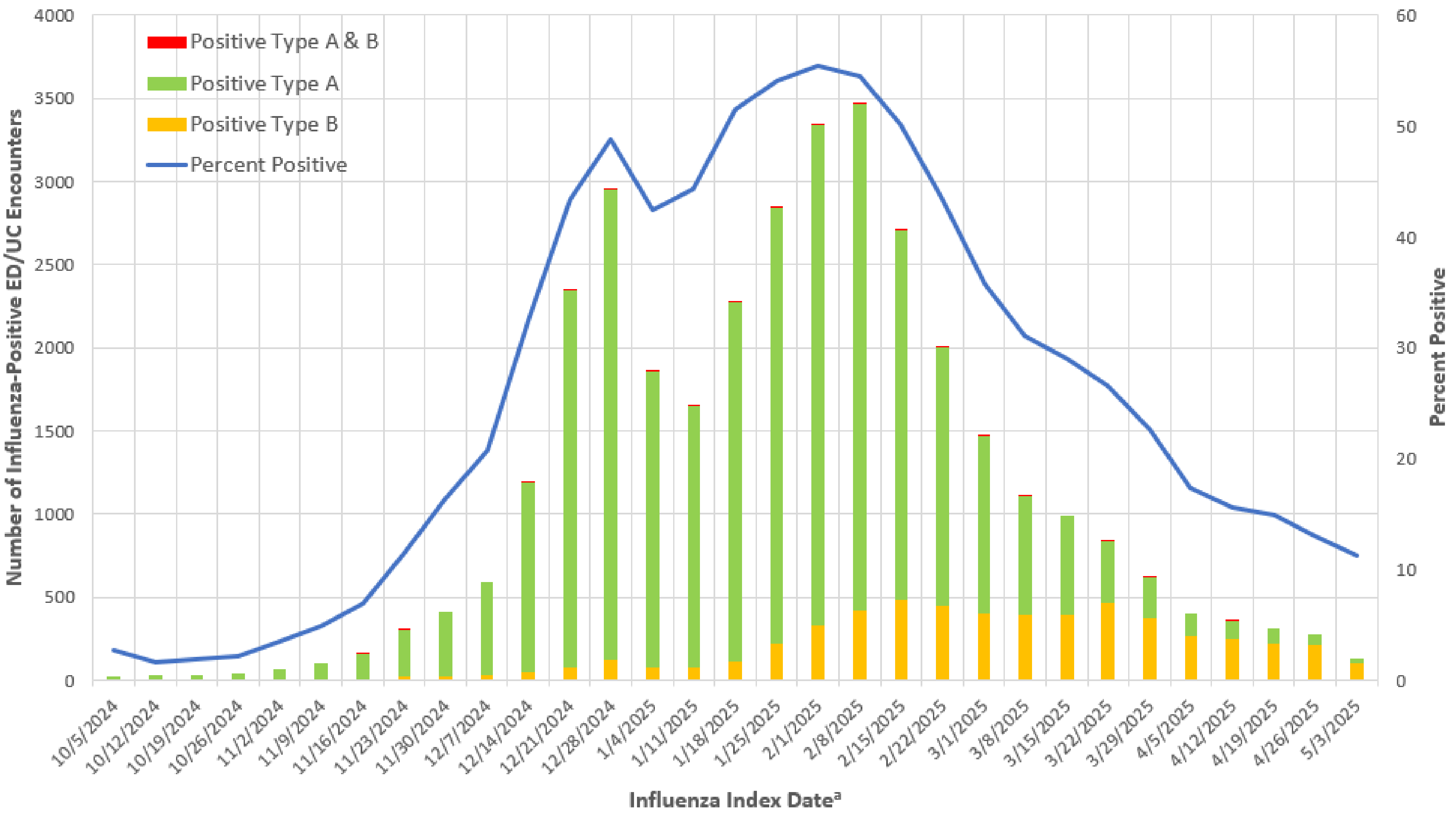

C) Influenza-positive hospitalizations and percent positivity by week among adults aged ≥18 years

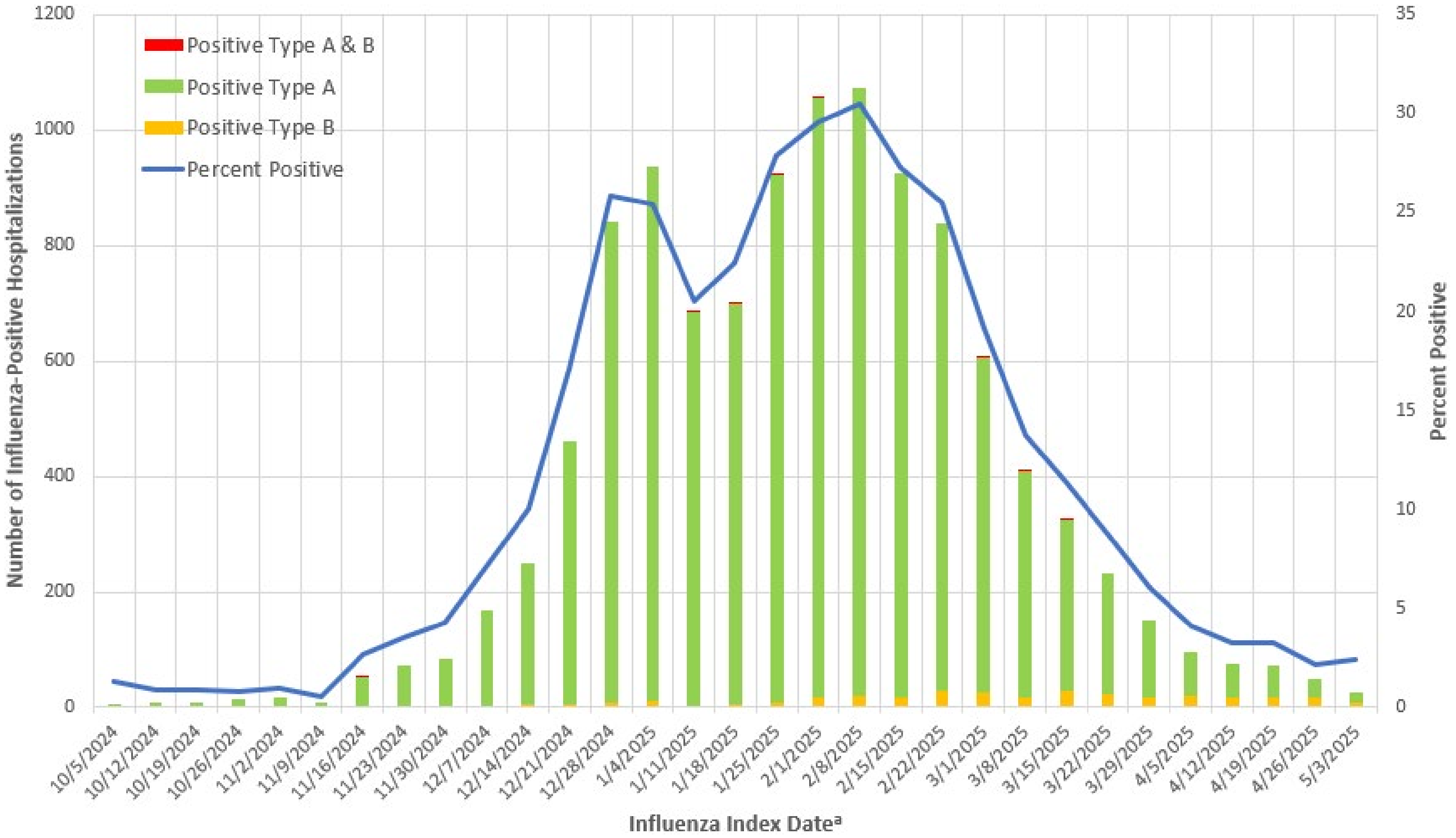

D) Influenza-positive emergency department or urgent care encounters and percent positivity by week among adults aged ≥18 years

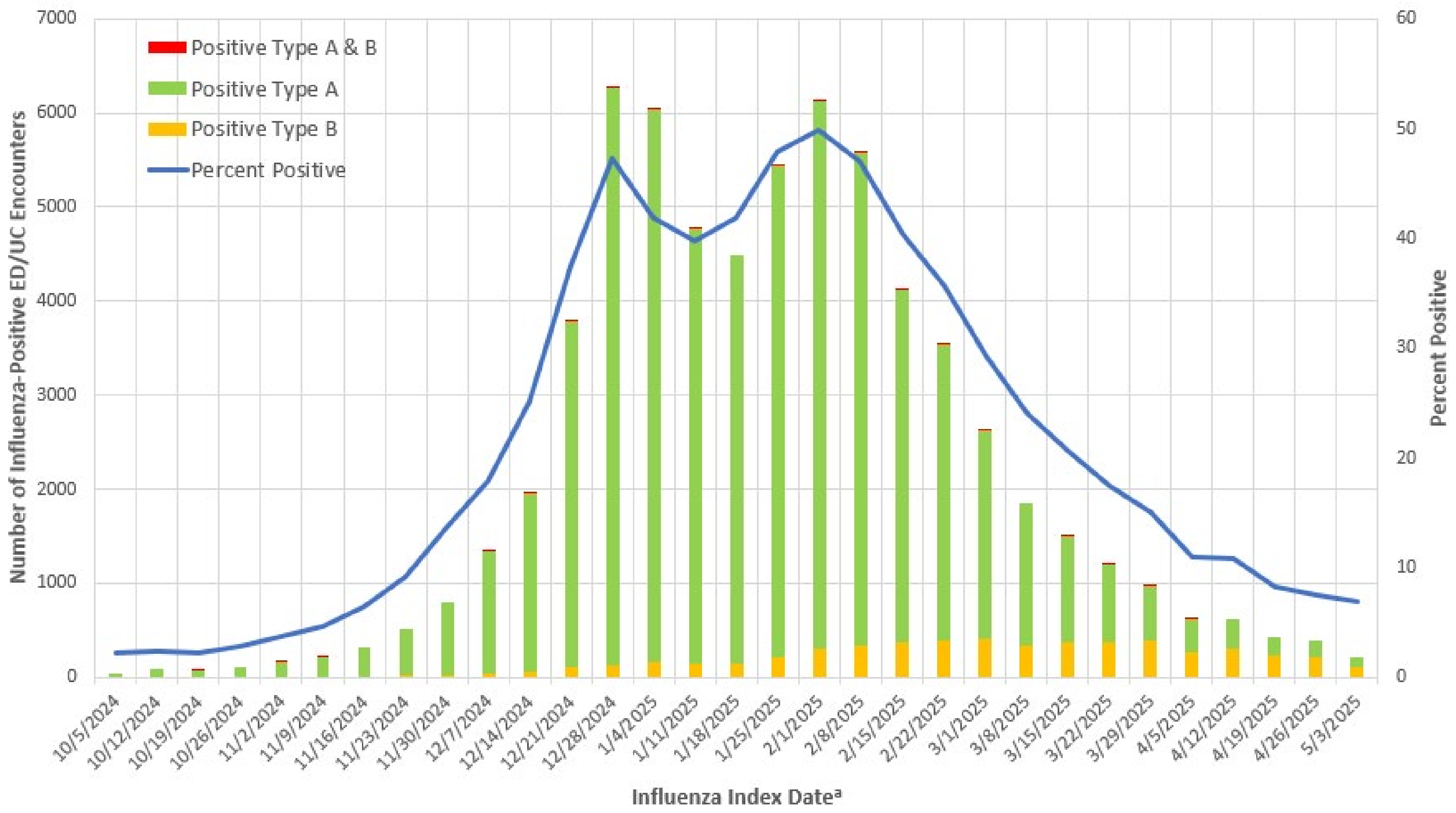

Abbreviations: ED/UC = emergency department or urgent care

[a] Influenza index date was defined as the earlier date of either the most recent influenza test or the encounter date.

Supplemental Table 4. Influenza vaccine product type received by site and age group — VISION, October 2024–April 2025[a]

	N (row %)[b]							N (row %)[c]
Age Group and Site	Live Attenuated (FluMist)	Standard-Dose Inactivated (Afluria, Fluarix, FluLaval, Fluzone)	Cell Culture-Based (Flucelvax)	Recombinant (Flublok)	High-Dose Inactivated (Fluzone High-Dose)	Adjuvanted (Fluad)	Other	Unknown/ Missing product type
Total	1,100 (0.8)	64,215 (44.3)	7,011 (4.8)	2,531 (1.7)	51,160 (35.3)	16,701 (11.5)	2,257 (1.6)	6,963 (4.6)
6 months–17 years	1,050 (4.0)	22,384 (84.5)	2,599 (9.8)	2 (0.0)	5 (0.0)	5 (0.0)	435 (1.6)	1,066 (3.9)
Site A	0 (0.0)	687 (99.6)	1 (0.1)	0 (0.0)	0 (0.0)	0 (0.0)	2 (0.3)	3 (0.4)
Site B	161 (10.7)	369 (24.6)	969 (64.5)	0 (0.0)	0 (0.0)	0 (0.0)	3 (0.2)	2 (0.1)
Site C	189 (5.1)	2,803 (75.5)	433 (11.7)	0 (0.0)	1 (0.0)	1 (0.0)	285 (7.7)	930 (20.0)
Site D	72 (2.0)	3,016 (82.8)	469 (12.9)	2 (0.1)	0 (0.0)	1 (0.0)	83 (2.3)	41 (1.1)
Site E	24 (1.0)	1,989 (84.4)	317 (13.4)	0 (0.0)	1 (0.0)	1 (0.0)	26 (1.1)	3 (0.1)
Site F	13 (0.2)	5,898 (93.4)	392 (6.2)	0 (0.0)	0 (0.0)	1 (0.0)	10 (0.2)	13 (0.2)
Site G	591 (7.2)	7,622 (92.3)	18 (0.2)	0 (0.0)	3 (0.0)	1 (0.0)	26 (0.3)	74 (0.9)
18–64 years	43 (0.1)	37,534 (83.7)	4,152 (9.3)	1,730 (3.9)	480 (1.1)	115 (0.3)	812 (1.8)	2,831 (5.9)
Site A	0 (0.0)	1,684 (94.6)	44 (2.5)	30 (1.7)	12 (0.7)	0 (0.0)	11 (0.6)	21 (1.2)
Site B	18 (0.6)	1,051 (35.9)	1,664 (56.8)	100 (3.4)	8 (0.3)	14 (0.5)	76 (2.6)	20 (0.7)
Site C	8 (0.3)	2,233 (72.2)	303 (9.8)	299 (9.7)	53 (1.7)	49 (1.6)	149 (4.8)	1,408 (31.3)
Site D	2 (0.0)	4,593 (69.8)	842 (12.8)	755 (11.5)	38 (0.6)	6 (0.1)	342 (5.2)	254 (3.7)
Site E	0 (0.0)	2,534 (60.9)	998 (24.0)	505 (12.1)	53 (1.3)	12 (0.3)	58 (1.4)	53 (1.3)
Site F	0 (0.0)	9,718 (97.2)	126 (1.3)	10 (0.1)	61 (0.6)	29 (0.3)	51 (0.5)	179 (1.8)
Site G	15 (0.1)	15,721 (96.3)	175 (1.1)	31 (0.2)	255 (1.6)	5 (0.0)	125 (0.8)	896 (5.2)
≥65 years	7 (0.0)	4,297 (5.8)	260 (0.4)	799 (1.1)	50,675 (68.8)	16,581 (22.5)	1,010 (1.4)	3,066 (4.0)
Site A	0 (0.0)	90 (2.6)	26 (0.7)	31 (0.9)	3,321 (94.7)	34 (1.0)	6 (0.2)	32 (0.9)
Site B	0 (0.0)	62 (1.2)	70 (1.4)	21 (0.4)	1,659 (32.9)	3,115 (61.8)	117 (2.3)	53 (1.0)

Site C	0 (0.0)	113 (2.7)	17 (0.4)	56 (1.3)	3,106 (74.8)	735 (17.7)	125 (3.0)	1,680 (28.8)
Site D	4 (0.1)	143 (2.9)	19 (0.4)	99 (2.0)	3,860 (77.1)	551 (11.0)	329 (6.6)	170 (3.3)
Site E	0 (0.0)	544 (6.1)	83 (0.9)	459 (5.2)	6,055 (68.0)	1,570 (17.6)	193 (2.2)	103 (1.1)
Site F	0 (0.0)	1,990 (7.8)	15 (0.1)	60 (0.2)	12,933 (50.5)	10,454 (40.8)	150 (0.6)	402 (1.5)
Site G	3 (0.0)	1,355 (6.3)	30 (0.1)	73 (0.3)	19,741 (92.2)	122 (0.6)	90 (0.4)	626 (2.8)

[a] Influenza vaccine product types are defined as follows: Live Attenuated (CVX 111), Standard-Dose Inactivated (CVX 140,141), Cell Culture-Based (CVX 153, 320), Recombinant (CVX 155), High-Dose Inactivated (CVX 135), Adjuvanted (CVX 168), Other (CVX 15, 16, 123, 125, 126, 127, 128, 144, 149, 150, 158, 160, 161, 166, 171, 185, 186, 194, 197, 200, 201, 202, 205, 231, 321, 322, 323), Unknown/Missing product type (CVX 88, 151, or missing/unknown).

[b] The denominators for these row percentages only include influenza vaccinations with a known vaccine product type.

[c] The denominators for these row percentages include all influenza vaccinations with known and unknown vaccine product types

Supplemental Table 5. Influenza vaccine effectiveness against influenza-associated hospitalizations and emergency department or urgent care encounters by influenza type, age group, and time since vaccination — VISION, October 2024–April 2025

	Hospitalizations[a]			Emergency Department or Urgent Care Encounters		
	Cases<br>Number vaccinated / total (%)	Controls<br>Number vaccinated / total (%)	Vaccine effectiveness[b]	Cases<br>Number vaccinated / total (%)	Controls<br>Number vaccinated / total (%)	Vaccine effectiveness[b]
**Influenza A**						
By age and time since vaccination						
**6 months–4 yrs**						
14-59 days	15/246 (6%)	324/2017 (16%)	63 (38-80)	483/8761 (6%)	3371/25992 (13%)	57 (52-61)
60-119 days	38/269 (14%)	415/2108 (20%)	52 (31-67)	904/9182 (10%)	4618/27239 (17%)	59 (56-63)
≥120 days	20/251 (8%)	405/2098 (19%)	54 (25-73)	489/8767 (6%)	3813/26434 (14%)	55 (50-60)
**5-17 yrs**						
14-59 days	17/351 (5%)	161/1614 (10%)	51 (17-73)	547/16383 (3%)	2194/27831 (8%)	60 (55-64)
60-119 days	41/375 (11%)	223/1676 (13%)	53 (31-68)	1724/17560 (10%)	2789/28426 (10%)	40 (36-44)
≥120 days	36/370 (10%)	265/1718 (15%)	18 (-23-47)	1001/16837 (6%)	2871/28508 (10%)	27 (21-34)
**18-49 yrs**						
14-59 days	47/1214 (4%)	529/6350 (8%)	54 (35-68)	795/28493 (3%)	4514/60723 (7%)	58 (55-62)
60-119 days	183/1350 (14%)	761/6582 (12%)	34 (19-47)	2639/30337 (9%)	6411/62620 (10%)	48 (45-51)
≥120 days	117/1284 (9%)	851/6672 (13%)	24 (3-42)	1628/29326 (6%)	6636/62845 (11%)	39 (35-43)
**50-64 yrs**						

14-59 days	79/1667 (5%)	1030/8653 (12%)	49 (34-61)	479/9880 (5%)	2981/22544 (13%)	58 (54-63)
60-119 days	310/1898 (16%)	1597/9220 (17%)	40 (30-48)	1878/11279 (17%)	4655/24218 (19%)	46 (42-49)
≥120 days	233/1821 (13%)	1863/9486 (20%)	40 (29-49)	1424/10825 (13%)	5298/24861 (21%)	40 (35-44)
**≥65 yrs**						
14-59 days	330/4067 (8%)	5670/24055 (24%)	56 (50-62)	711/7707 (9%)	8175/30020 (27%)	59 (55-62)
60-119 days	1515/5252 (29%)	8995/27380 (33%)	47 (42-51)	3705/10701 (35%)	13727/35572 (39%)	45 (42-48)
≥120 days	1526/5263 (29%)	11769/30154 (39%)	36 (31-41)	3309/10305 (32%)	17098/38943 (44%)	35 (32-39)
**Influenza B**						
By age and time since vaccination						
**6 months – 4 yrs**						
14-59 days	1/23 (4%)	324/2017 (16%)	--	15/733 (2%)	3371/25992 (13%)	82 (71-90)
60-119 days	1/23 (4%)	415/2108 (20%)	--	26/744 (3%)	4618/27239 (17%)	85 (78-90)
≥120 days	1/23 (4%)	405/2098 (19%)	--	30/748 (4%)	3813/26434 (14%)	87 (82-92)
**5-17 yrs**						
14-59 days	1/53 (2%)	161/1614 (10%)	--	60/4193 (1%)	2194/27831 (8%)	77 (69-83)
60-119 days	5/57 (9%)	223/1676 (13%)	--	154/4287 (4%)	2789/28426 (10%)	75 (71-80)
≥120 days	9/61 (15%)	265/1718 (15%)	60 (18-82)	474/4607 (10%)	2871/28508 (10%)	54 (49-59)
**18-49 yrs**						
14-59 days	7/155 (5%)	529/6350 (8%)	49 (-9-80)	41/4254 (1%)	4514/60723 (7%)	81 (74-86)
60-119 days	11/159 (7%)	761/6582 (12%)	67 (40-84)	186/4399 (4%)	6411/62620 (10%)	68 (63-72)

≥120 days	21/169 (12%)	851/6672 (13%)	65 (42-79)	365/4578 (8%)	6636/62845 (11%)	67 (63-71)
**50-64 yrs**						
14-59 days	2/40 (5%)	1030/8653 (12%)	--	12/424 (3%)	2981/22544 (13%)	69 (48-84)
60-119 days	1/39 (3%)	1597/9220 (17%)	--	21/433 (5%)	4655/24218 (19%)	81 (72-88)
≥120 days	10/48 (21%)	1863/9486 (20%)	--	75/487 (15%)	5298/24861 (21%)	67 (57-75)
**≥65 yrs**						
14-59 days	3/58 (5%)	5670/24055 (24%)	69 (29-90)	7/147 (5%)	8175/30020 (27%)	73 (49-87)
60-119 days	8/63 (13%)	8995/27380 (33%)	76 (55-88)	25/165 (15%)	13727/35572 (39%)	70 (53-82)
≥120 days	30/85 (35%)	11769/30154 (39%)	60 (38-75)	85/225 (38%)	17098/38943 (44%)	65 (53-74)

[a] Vaccine effectiveness estimates are not shown against influenza B-associated hospitalizations for some groups due to case counts <50 or confidence interval widths ≥100 percentage points.

[b] Vaccine effectiveness was estimated using logistic regression models comparing the odds of vaccination between influenza-positive cases and influenza-negative controls. Models were adjusted for age, sex, race and ethnicity, calendar day, and site, with age and calendar day treated as natural cubic splines with 4 degrees of freedom.
